# Supplementary material for: Quantifying the thermodynamics of protein unfolding using 2D NMR spectroscopy
Source: Commun Chem. 2020 Aug 7;3:100. doi: 10.1038/s42004-020-00358-1 (PMC7116895; doi:10.1038/s42004-020-00358-1)
Supplement: Supplementary file 2 — Supplementary Information [file 42004_2020_358_MOESM2_ESM.pdf]

Supplementary Information

Quantifying the thermodynamics of protein unfolding using 2D NMR spectroscopy

Rita Puglisi[a], Oliver Brylski[b], Caterina Alfano[c], Stephen R. Martin[d], Annalisa Pastore[a], Piero A. Temussi[a] ed]*

[a] King's College London, The Wohl Institute, 5 Cutcombe Rd, SE59RT London (UK)

[b] Institute of Physical and Theoretical Chemistry, Technische Universität Braunschweig, Braunschweig, Germany.

[c]Fondazione Ri.Med, Palermo, 90133, Italy

[d] The Crick Intstute, 1 Midland Rd, London NW1 1ST, United Kingdom

[e] Dipartimento di Scienze Chimiche, Universita' di Napoli Federico II, Napoli, Italy.

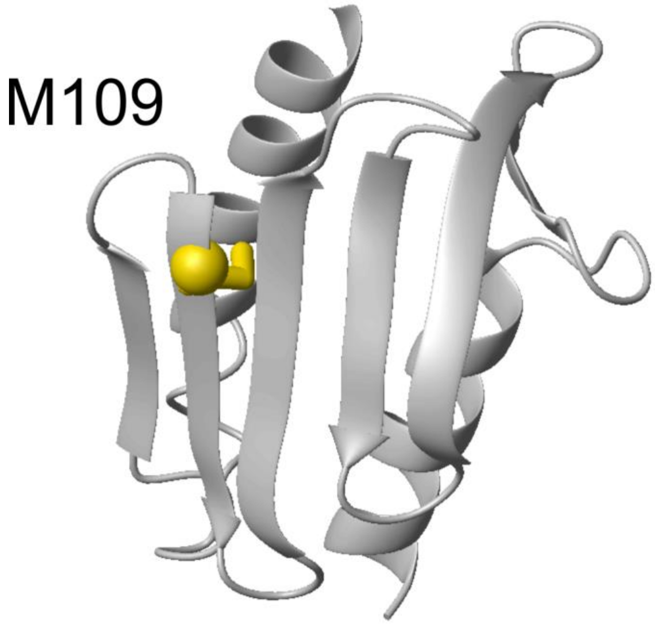

Supplementary Figure 1 – Residue exposure. Ribbon representation of the structure of Yfh1 showing the relative positions of the sidechain of Met 109 (gold neon) and its NH group (gold ball). The sidechain is completely buried between the beta sheet and a helix, as shown by the zero residue SASA (Table S1) whereas the NH group is exposed to the solvent. The N terminal disordered trait of the Yfh1 structure is not shown. The model was built using MOLMOL [1].

Supplementary Table 1. List of isolated and clearly identifiable resonances of the amide resonances with the corresponding parameters calculated by Pops for the X-ray structure of a mutant of Yfh1 (2flq). Notice that this structure does not contain the last two C-terminal residues which are clearly identifiable in the spectrum. They are anyway fully exposed. Residues are color coded according to their RAD values as detailed below.

			**D[a]**	**SASA[b]/residue**	**SASA[c/] atom**	**RA[d]**	**D x RA x 100 = RAD**
ATOM	VAL	61	1.56	207	33.76	0.309	48.2
ATOM	GLN	63	1.04	148	3.86	0.035	3.64
ATOM	GLU	64	0.97	161	2.81	0.026	2.52
ATOM	VAL	65	0.83	69	0.41	0.0037	0.31
ATOM	LEU	68	0.98	93	0.84	0.0077	0.75
ATOM	LEU	70	1.12	97	2.25	0.021	2.35
ATOM	GLU	71	1.15	177	6.78	0.062	7.13
ATOM	LYS	72	1.20	181	5.67	0.052	6.24
ATOM	GLU	75	0.74	70	3.10	0.028	2.07
ATOM	GLU	76	0.61	102	1.66	0.015	0.91
ATOM	ASP	78	0.32	49	0.57	0.0052	0.17
ATOM	ASP	86	0.56	91	0.50	0.0045	0.27
ATOM	SER	87	0.68	49	0.55	0.005	0.34
ATOM	LEU	88	0.39	18	0.19	0.0018	0.039
ATOM	GLU	89	0.34	88	0.64	0.0059	0.20
ATOM	GLU	90	0.65	130	0.88	0.0081	0.52
ATOM	LEU	91	0.61	65	0.30	0.0027	0.16
ATOM	SER	92	0.46	22	0.36	0.0033	0.15
ATOM	GLU	93	0.76	125	0.89	0.008	0.61
ATOM	ALA	94	0.94	70	4.81	0.044	4.1
ATOM	HIS	95	0.63	42	0.48	0.0044	0.28
ATOM	ASP	97	1.02	138	1.02	0.0093	0.95
ATOM	CYS	98	0.69	25	0.40	0.0037	0.26
ATOM	ILE	99	0.31	15	0.40	0.0037	0.11
ATOM	ASP	101	0.55	62	2.15	0.0197	1.08
ATOM	LEU	104	0.59	43	1.45	0.0132	0.78
ATOM	SER	105	0.75	79	1.84	0.0168	1.18
ATOM	GLY	107	0.73	20	5.36	0.049	3.58
ATOM	VAL	108	0.55	53	1.01	0.0092	0.50
ATOM	MET	109	0.30	0	2.29	0.021	0.63
ATOM	THR	110	0.23	36	1.22	0.011	0.23
ATOM	GLU	112	0.32	89	1.43	0.0131	0.41
ATOM	ILE	113	0.34	0	0.38	0.0035	0.12
ATOM	ALA	115	0.84	67	3.22	0.0295	2.48
ATOM	PHE	116	0.70	60	0.97	0.0089	0.62
ATOM	GLY	117	0.75	23	1.43	0.013	0.98
ATOM	TYR	119	0.33	4	0.73	0.0067	0.22
ATOM	VAL	120	0.38	44	0.95	0.0087	0.33
ATOM	ASN	127	1.14	183	5.55	0.051	5.81
ATOM	LYS	128	0.83	84	0.47	0.0081	0.66
ATOM	GLN	129	0.35	47	0.63	0.0057	0.2
ATOM	ILE	130	0.05	0	0.48	0.0044	0.02
ATOM	TRP	131	0.07	76	0.59	0.0054	0.04
ATOM	LEU	132	0.10	1	0.22	0.0020	0.02
ATOM	ALA	133	0.28	30	0.74	0.0067	0.19
ATOM	SER	134	0.33	3	0.43	0.0039	0.13
ATOM	LEU	136	0.68	92	0.40	0.0037	0.25

ATOM	ASN	140	0.40	19	0.47	0.0043	0.17
ATOM	PHE	142	0.06	0	0.51	0.0047	0.03
ATOM	ASP	143	0.22	57	0.65	0.0059	0.13
ATOM	ASN	146	1.07	151	2.03	0.0186	2.0
ATOM	GLY	147	1.10	57	4.82	0.044	4.8
ATOM	GLU	148	0.79	62	1.95	0.0179	1.4
ATOM	VAL	150	0.16	31	0.24	0.0022	0.03
ATOM	SER	151	0.28	4	0.21	0.0019	0.05
ATOM	LEU	152	0.40	77	0.45	0.0041	0.16
ATOM	ASN	154	0.95	112	1.36	0.012	1.14
ATOM	LEU	158	0.17	2	0.20	0.0018	0.03
ATOM	THR	159	0.25	37	0.39	0.0035	0.09
ATOM	ASP	160	0.54	69	0.56	0.0051	0.28
ATOM	ILE	161	0.42	23	0.24	0.0022	0.09
ATOM	THR	163	0.34	17	0.47	0.0044	0.15
ATOM	VAL	166	0.17	24	0.36	0.0033	0.06
ATOM	LYS	168	0.56	87	0.32	0.0029	0.16
ATOM	ILE	170	0.47	67	0.73	0.0067	0.31
ATOM	LYS	172	1.00	228	1.59	0.015	1.5

[a]The depth index (D) for an atom i and a sampling radius *r*, is defined as $D_{i,r}=2V_{i,r}/V_{0,r}$, where $V_{i,r}$ is the exposed volume of a sphere of radius r centered on atom i and $V_{0,r}$ is the exposed volume of the same sphere when centered on an isolated atom. [2]

[b] Solvent Accessible Surface Area per residue as furnished by DSSP [3]

[c] Solvent Accessible Surface Area per atom as furnished by [4]

[d] Relative accessible area for a given atom, corresponding to parameter Q(SASA) of PopS [4]

Color code
N with **RAD** between 0.4 and 0.5
N with **RAD** between 0.3 and 0.4
N with **RAD** between 0.2 and 0.3
N with **RAD** between 0.1 and 0.2
N with **RAD** between 0.0 and 0.1

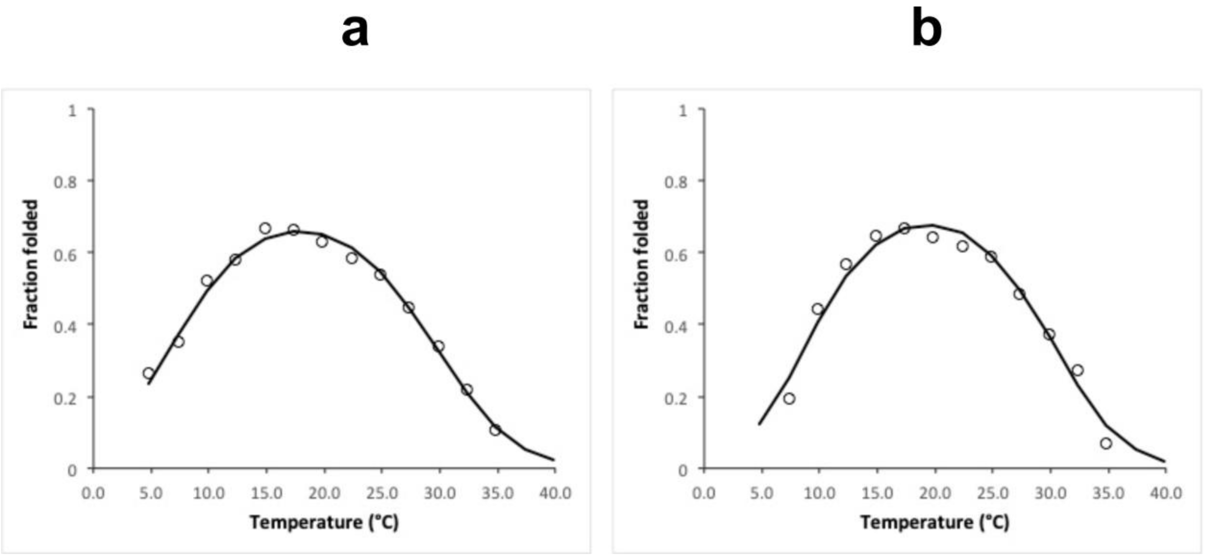

Supplementary Figure 2 – Fitting procedure. Two plots of folded fractions of Yfh1 as a function of temperature related to Figures 2 and 3. **a**) Fitting of folded populations derived from relative volumes of all corrected resonances of Figure 2. The non-linear fitting utilized the modified Gibbs Helmholtz equation (see main text). **b**) Fitting of folded populations derived from relative volumes of non-overlapping resonances with RAD< 0.1 of Figure 3.

Supplementary References

1. Koradi, R., Billeter, M. & Wüthrich, K. MOLMOL: a program for display and analysis of macromolecular structures. *J. Mol. Graph*. **14**, 51-5. (1996)

2. Varrazzo, D., Bernini, A., Spiga, O., Ciutti, A., Chiellini, S., Venditti, V., Bracci, L. & Niccolai, N. Three-dimensional computation of atom depth in complex molecular structures. *Bioinformatics* **21**, 2856-60 (2005).

3. Kabsch, W. & Sander, C. Dictionary of protein secondary structure: pattern recognition of hydrogen-bonded and geometrical features. *Biopolymers*. 22, 2577-637 (1983).

4. Cavallo, L., Kleinjung, J. & Fraternali, F. POPS: A fast algorithm for solvent accessible surface areas at atomic and residue level. *Nucleic Acids Res*. **31**, 3364-6 (2003).
